# Assessment of quality of data provided on Pap test requisitions: Implications for quality of care and patient safety

**DOI:** 10.4103/1742-6413.53360

**Published:** 2009-07-11

**Authors:** Sonya Naryshkin, Brenda L. Schultz

**Affiliations:** Mercy Hospital Laboratory, 1000 Mineral Point Ave., PO Box 5003, Janesville, WI 53547, USA

**Keywords:** Pap test, requisitions, quality assurance, pre-analytical, high-risk, history, clinical information

## Abstract

**Background:**

The reliability of patient history and clinical information on Pap test requisitions has been questioned but not previously objectively determined. The effect of incomplete/inaccurate information on quality of patient care has not been previously quantified. Our objectives were (1) to find out how clinicians and their assistants viewed the requisition slip, and whether they understood the reasons for supplying the information requested, (2) to measure the completeness and accuracy of information on the requisition slips, and (3) to determine whether the clinical information and patient history provided on Pap test requisitions could be relied upon to accurately assign a Pap test to the laboratory's “high-risk rescreen” pool.

**Methods:**

Clinicians and their assistants were surveyed. A total of 899 consecutive Pap test requisition slips were reviewed. Patient history and clinical information from the slips were compared to data from our laboratory information system and/or electronic patient medical records.

**Results:**

Most survey respondents felt that proper completion of requisitions was important, but only 17% of clinicians and less staff realized that negative high-risk Pap tests underwent a quality assurance rescreen. Clinicians and/or staff recorded the last menstrual period, specimen source, and clinical information on the requisition slips 96%, 97%, and 88% of the time, respectively. Of 695 Pap tests with applicable computerized records, 172 (25%) qualified for high-risk rescreen based upon information provided on the requisition slip alone. An additional 52 Pap tests (7%), or 23% of the total high-risk Pap tests were discovered to be of high risk only after review of the electronic records.

**Conclusions:**

Clinicians and staff were receptive to discussions concerning the completion of requisition slips, but laboratory expectations could be better communicated. Requisition slips were properly completed with a high frequency, but the check boxes did not elicit all the information expected, so revision was necessary. The high accuracy of the completion of requisition slips permitted 77% of high-risk Pap tests to be identified via the requisition slip alone. Our findings challenge the conventional anecdotal impressions of “notoriously unreliable” information on Pap test requisition slips, but our experience may not be applicable to other settings.

## BACKGROUND

The Pap test requisition serves as a tool of communication between the laboratory and the submitting physician or authorized paramedical personnel such as nurse practitioners and physician assistants. As such, the Clinical Laboratory Improvement Amendments (CLIA '88) require laboratories to solicit certain patient identification and clinical information[1] as would be appropriate for any other medical consultation. Still, anecdotal reports indicate that the information supplied on Pap test requisition slips is often incomplete or inaccurate. To our knowledge, this relatively common anecdotal impression has not been studied or documented in any systematic way. We could find no references in the peer-reviewed medical literature that addressed the question of accuracy of information supplied on the Pap test requisition slip. To our knowledge, there are no published data on how the reliability of data supplied on Pap test requisitions might affect patient safety.

The objectives of our study were threefold. The first was to find out what clinicians and their staff knew, and what their attitudes were, concerning the significance of providing the laboratory with the patient information requested via the requisition slip. The second objective was to measure the completeness and accuracy of information supplied on the Pap test requisitions. The third was to determine the extent to which incomplete/inaccurate patient history and clinical information provided on Pap test requisition slips might affect the quality of patient care. Specifically, we asked whether the information supplied on the Pap test requisitions could be relied upon to accurately assign the high-risk Pap tests to our laboratory's high-risk rescreen pool.

Preliminary results of the surveys and a subset of the case reviews were presented in a poster session at the annual meeting of the College of American Pathologists (CAP '07) in Chicago, IL, on October 1, 2008 and in the subsequently published abstract.[2]

## MATERIALS AND METHODS

Our health system is a vertically integrated health care system comprising a network of 3 hospitals and 31 outlying clinics in two states. Our laboratory processed 21,374 Pap tests in 2006.

To address the first objective, we designed two surveys: one for clinicians (physicians, nurse practitioners, and physician's assistants) [Table 1] and a modified one for staff (nurses, medical assistants, others) [Table 2]. We identified the clinic/physician sources of Pap tests we received. We contacted each by telephone, in person, or by interoffice mail. We distributed the surveys to all physicians, nurse practitioners, physician assistants, resident physicians, medical students on applicable rotations, nurses, and medical assistants who we could identify as being involved in filling out or submitting Pap test requisitions. We distributed 110 clinician surveys and 116 staff surveys. We tabulated the results.

**Table 1:** Pap test requisition survey for clinicians

*Check all boxes that reflect your current view/practice*				
How do you view the completion of clinical information and clinical history on the Pap test requisition slip? It is important to convey pertinent information to the lab in order to receive the best care for my patient. It is required for regulatory compliance and billing. It is merely busywork and has minimal affect on the outcome of the pap result. Does your cytology laboratory treat pap tests differently based upon clinical information and history you provide on the requisition? I don't know/not sure High-risk patients are subject to a second review if the Pap is negative. Cytotechnologists and pathologists use the information to interpret cellular changes in a particular patient, but the screening protocol is the same for all. No, the information is important for billing and regulatory compliance only. Who fills out the clinical information, LMP, source of specimen and clinical history sections of the requisition? I do. My nurse/assistant while seeing the patient and viewing the chart prior to my arrival in the room. My nurse/assistant who is in the room with me at the time of collection does. Someone else (who is not in the room at the time the specimen is taken) fills it out. How is the clinical information and patient history that is recorded on the requisition slip gathered? From the patient's chart. By asking the patient questions and recording the responses. The clinician performing the exam provides the information. The assistant gathers the information. I do not know. Is the patient clinical information and history readily and easily retrievable? Yes No It is important for the lab to know if a patient has had HSIL and/or a cone biopsy or cryotherapy in the past Year Two years Even if 10 years ago The lab uses the “specimen source” information To correlate with cytologic findings. To determine adequacy of the specimen. To correlate with clinical information and history. All of the above. Our requisition slip includes a space for “specimen source.” How do you determine the source? Cervical/endocervical is usually checked automatically in cyclic women and women without a hysterectomy. Vaginal is usually checked automatically in women with a hysterectomy. Cervix is only checked if the clinician visualizes the cervix during specimen collection. Cervix and/or cervix/endocervical is checked for patients with a supracervical hysterectomy. The source is checked prior to the exam. The source is checked after the exam. The source is checked after the patient and clinician are gone.				
Completed by:	□ Staff Physician	□ Nurse Practitioner	□ Physicians Assistant	□ Resident

**Table 2:** Pap test requisition survey for staff

*Check all boxes that reflect your current view/practice*			
How do you view the completion of clinical information and clinical history on the Pap test requisition slip? It is important to convey pertinent information to the lab in order to receive the best care for the patient. It is required for regulatory compliance and billing. It is merely busywork and has minimal affect on the outcome of the pap result. Does your cytology laboratory treat pap tests differently based upon clinical information and history you provide on the requisition? I don't know/not sure High-risk patients are subject to a second review if the Pap is negative. Cytotechnologists and pathologists use the information to interpret cellular changes in a particular patient, but the screening protocol is the same for all. No, the information is important for billing and regulatory compliance only. Who fills out the clinical information, LMP, source of specimen and clinical history sections of the requisition? I do. The clinician collecting the Pap test completes the information Someone else (who is not in the room at the time the specimen is taken) fills it out. How is the clinical information and patient history that is recorded on the requisition slip gathered? From the patient's chart. By asking the patient questions and recording the responses. The clinician performing the exam provides the information. The assistant gathers the information. I do not know. Is the patient clinical information and history readily and easily retrievable? Yes No It is important for the lab to know if a patient has had HSIL and /or a cone biopsy or cryotherapy in the past Year Two years Even if 10 years ago The lab uses the “specimen source” information To correlate with cytologic findings. To determine adequacy of the specimen. To correlate with clinical information and history. All of the above. Our requisition slip includes a space for “specimen source”. How do you determine the source? Cervical/endocervical is usually checked automatically in cyclic women and women without a hysterectomy. Vaginal is usually checked automatically in women with a hysterectomy. Cervix is only checked if the clinician visualizes the cervix during specimen collection. Cervix and/or cervix/endocervical is checked for patients with a supracervical hysterectomy. The source is checked prior to the exam. The source is checked after the exam. The source is checked after the patient and clinician are gone. When is the patient identifier (name or label) put on the specimen? Prior to the patient arrival in the room. After patient arrival but prior to specimen collection. At the time of specimen collection. After the patient and clinician have left the room.			
Completed by:	□ Nurse	□ Medical assistant	□ Other:___________

To measure the completeness and accuracy of the information supplied on the requisition slips, we retrospectively reviewed 899 consecutive Pap test requisitions accessioned between May 12, 2006 and May 30, 2006. We devised an Excel spreadsheet for data collection. For each requisition, we collected two sets of data. The first set was obtained from the Pap test requisition itself and included specimen source, last menstrual period (LMP), clinical information, and patient history. The source was recorded as “cervical/endocervical,” “vaginal,” or “not given.” The LMP was recorded as “yes” or “no.” If the requisition slip indicted that the patient was pregnant, postpartum, postmenopausal, or had a hysterectomy, we considered that the question of “LMP” was answered. The clinical information that we looked for was pregnant, postpartum, postmenopausal, hormone therapy, abnormal bleeding, discharge, clinically high-risk, “none indicated,” and “none given.” “None given” meant that no boxes were checked and no information was written on the slip. “None indicated” meant that the slip had the null sign or “none” or “none pertinent” written on the slip, indicating that the questions were considered and answered. The patient history items we sought were history of dysplasia, cancer, or high-risk human papillomavirus infection (HPV), previous abnormal Pap test, supracervical hysterectomy, total hysterectomy, “none indicated,” and “none given”. We then determined if the patient fulfilled our laboratory's criteria for “high-risk” quality assurance rescreen [Table 3] based upon that information (yes or no). When the patient was determined to be high-risk based upon the information provided on the requisition slip, we recorded one of the following reasons for the clinically high-risk indication: abnormal bleeding, history of dysplasia, HPV, cancer, or prior abnormal Pap, or a combination thereof.

**Table 3:** Laboratory criteria for triage to high-risk rescreen

Previous Pap with history of ASCUS (aypical squamous cells of undetermined significance)/AGUS (atypical glandular cells of undetermined significance): If current Pap negative, QC (quality control re-screen) is required (one time) If HPV (high risk Human Papillomavirus) test is (−), no QC is required If HPV test is (+), QC required until 3 consecutive negative yearly Paps Clinical information of previous abnormals elsewhere: QC required until 3 consecutive negative yearly Paps (regardless of how many years ago) Previous Pap is LSIL (low grade squamous intraepithelial lesion) or above: QC required until 3 consecutive negative yearly Paps History of DES (diethylstilbestrol) exposure Patient clinical information or history includes a (+) HPV test: QC required until 3 consecutive negative yearly Paps Patient history or clinical information indicates no Pap smear in past 5 years Clinical information of abnormal bleeding Clinical information of High Risk History of other GYN (gynecologic) malignancies, i.e. ovary

The second set of data was obtained from checking our computerized laboratory information system (LIS) history card file (STAR AP Laboratory Application, McKesson Corporation, San Francisco, CA, USA) and also by checking our health system's computerized patient record system, Chartview (SoftMed Systems, Inc., Silver Spring, MD, USA). The history card file contains all cytology and histology results reported by our laboratory. Chartview contains records of patient visits and procedure notes, either written or audio (dictated). If a particular patient did have LIS or Chartview records available but there were no entries related to the gynecologic (GYN) system or other pertinent records (such as history of cancer of another body system, immunosuppression, etc.), we counted that as no records.

Based upon the computerized data only, we again determined whether or not the patient met our laboratory definition of “high-risk” for the purpose of triggering a second, quality assurance slide screen if the initial one was negative. We compared the determinations of high-risk status from the two sets of data. We especially looked at the number of instances that a patient, not considered “high-risk” by the information on the test requisition, did meet the criteria for “high-risk” rescreen when the additional information from the history card file or Chartview was reviewed. These are patients whose Pap slides would have been denied a rescreen if their initial screen had been negative and if we had not had the additional computerized information.

## RESULTS

Of the 110 clinician surveys distributed, 46 were returned. Of the 116 staff surveys distributed, 47 were returned. Almost all (95% of clinicians and 96% of staff) respondents agreed with the statement “it is important to convey pertinent information to the lab in order to receive the best care for their patients.” And, almost all respondents reported that the clinical information and history were readily available, although slightly less staff than clinicians thought so. In contrast to 9% of the clinicians, none of the staff viewed the requisition slip questions, as mere busywork.

Notably, 63% of clinicians and 53% of their staff answered that they did not know or were not sure if or how the laboratory treated the Pap tests based upon the clinical information and history they supplied. Specifically, only 17% of clinicians and 14% of staff knew that we in the laboratory subjected high-risk Pap tests to a second screen.

The vast majority of clinicians (74%) reported that their nurses or assistants completed the clinical information, LMP, and specimen source information while seeing the patient and viewing the chart prior to the arrival of the clinician in the room. The staff surveys corroborated that. The clinical information recorded on the requisition slip was reportedly gathered by asking the patient most of the time, from the chart about half the time, and from information relayed by the clinician for the remainder. Seventy-four percent of all respondents thought that it was important for the lab to know about prior high-grade squamous intraepithelial lesion (HSIL), cone biopsy, or cryotherapy in the past. Most respondents recognized that the lab used “specimen source” to correlate with cytologic findings, determine specimen adequacy, and to correlate with clinical information and history. A variety of protocols were used to determine the “specimen source.” Fifty-four percent of respondents usually automatically checked the “cervical/endocervical” box for patients who were cyclic and for those without a hysterectomy. Thirty percent reported automatically checking the “vaginal” box for women with a history of hysterectomy, and slightly less reported checking the “cervix” and or cervix/endocervical” for a woman with a supracervical hysterectomy.

**Figure 1:**
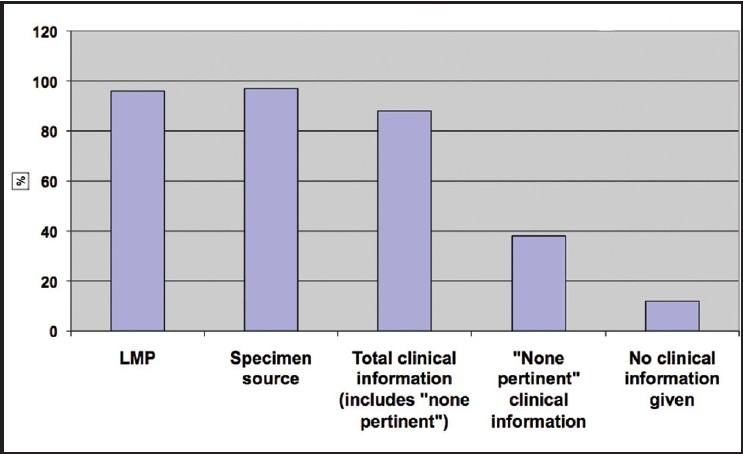
Frequency of information provided on Pap test requisition slips

**Figure 2:**
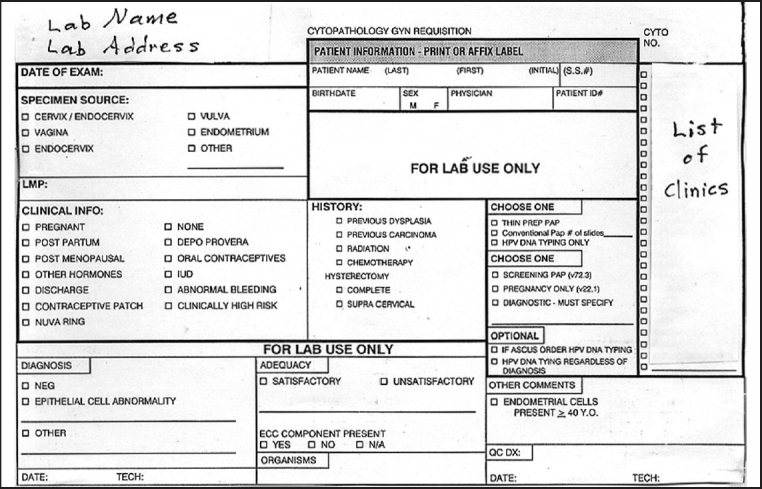
Original Pap test requisition slip

**Figure 3:**
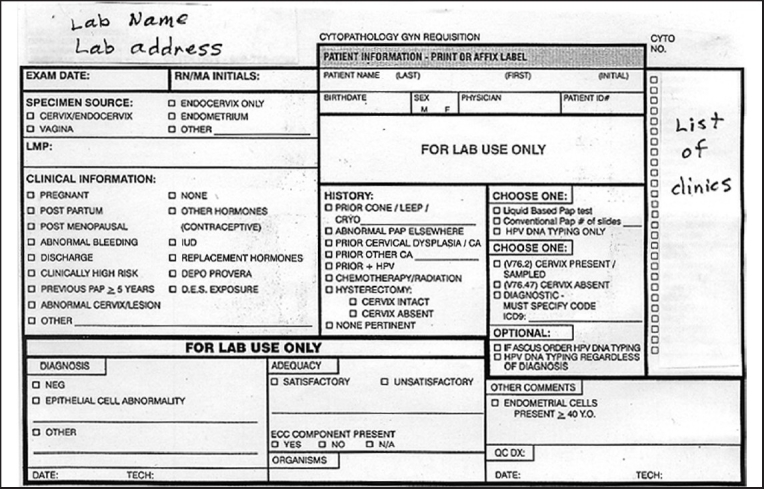
Revised Pap test requisition slip. Multiple additions, deletions, and modifications were made to the “specimen source,” “clinical information,” and “history” categories (compare to original requisition slip in Figure 2)

Figure 1 shows the frequency of information provided on the requisition slips. During our initial pilot phase of the project, which was reported in the previously published abstract,[2] we discovered that the check boxes on our requisition slips then in use [Figure 2] did not elicit all the information we expected and that we needed in order to triage a Pap case to our high-risk rescreen pool. Therefore, we redesigned our requisition slip mid-project [Figure 3] making sure to provide a check box for each of the high-risk criteria chosen by our laboratory.

The responses to our check boxes and queries on the requisition slips were tabulated. LMP and specimen source were given in 96% and 97% of cases, respectively. Clinical information was given in 88% of cases. Within this group, there was an indication of “no clinical information,” i.e., the question was considered and answered, in 38 % of the total cases. There was no clinical information given at all in 12% of cases.

**Figure 4:**
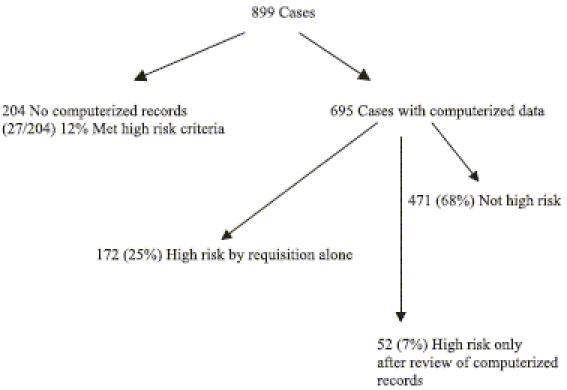
Flow chart of analysis of Pap test requisition slip data

A flow sheet of our analysis of the requisition slip data is shown in Figure 4. Of the 899 cases, there were 205 or 23% for whom we did not have any pertinent computerized LIS or Chartview records. Twenty-seven or 12% of these 205 patients were determined to meet our laboratory's high-risk criteria based upon the requisition slips only.

Further study was limited to the 695 or 77% of total cases who did have applicable and pertinent computerized records. Of these latter, 172/695 or 25% met our laboratory's high-risk criteria based upon the data on the requisition slips alone. Review of the computerized records confirmed the high-risk status in 146 of these 172 patients (85%). In 26 other, high-risk boxes were checked on the requisition (such as HPV+) but upon further review we found that these cases did not actually meet our specific laboratory triggers for a high-risk rescreen. For example, a patient might have had a positive HPV test in the remote past, but the infection had since cleared and that patient had subsequently three consecutive, yearly “no intraepithelial lesion or malignancy” Pap tests and a negative follow-up HPV test. Thus, such a patient's Pap would not have been triaged for a high-risk rescreen. However, we kept these cases in the “high-risk” pool for calculations since if we had only the requisition slips to rely upon, they would have been treated as such. In addition, if the requisition box “clinically high-risk” was checked, we always considered that Pap “high-risk” no matter what the computerized records showed, as the clinician might have had additional information that was not available to us.

Of the 695 requisition slips with applicable computerized records, an additional 52 cases or 7% of this group were discovered to meet our laboratory's high-risk criteria only after examination of the computerized records. The requisition slips, when viewed independently, lacked the pertinent information that would have alerted us to the high-risk status of these patients. This group of potentially misclassified patients accounted for 23% (52/224) of the total high-risk group.

Further study of the group of 52 high-risk patients identified only by computerized records revealed surprising findings. For 35 of them (67%), the requisition slips had no clinical history given at all. In nine other cases, the person filling out the requisition slip actually wrote the “null” sign or the words “none” in the section for pertinent clinical history.

For the 52 patients who we reclassified as “high-risk,” the reasons for the reclassification were HPV+ (5), clinically high-risk (4), abnormal bleeding (9), prior abnormal Pap (15), and dysplasia (24). Some patients had more than one reason for the reclassification.

## DISCUSSION

As far as we know, this is the first reported measurement of baseline accuracy of patient history and clinical information supplied on Pap test requisition slips. In the USA, CLIA '88 § 493.1241 spells out that “For Pap smears, the patient's last menstrual period, and indication of whether the patient had a previous abnormal report, treatment, or biopsy”[1] is to be solicited by the laboratory to be supplied with the Pap test specimen. However, only the lack of patient identifiers such as patient name is a reason for rejection of the specimen under the regulations. As a result, some Pap tests will be processed despite the absence of significant clinical information or patient history.

“The importance of supplying pertinent clinical information in Pap smear requisitions…seems to have gone largely unrecognized, despite the educational efforts of the American College of Obstetrics and Gynecologists, American Academy of Family Practice, and American College of Physicians.”[3] The probability that lack of such pertinent information will lead to less than optimal patient outcomes is accentuated by the highly fragmented system of health care currently in the USA. It may be an understatement to say that most Pap tests performed in the USA are processed in a laboratory that is remote from the procuring clinician and patient, and that the laboratory may not have regular physical or telephone contact with the clinician or access to patient charts. Informal regular person-to-person discussions between pathologists and other clinicians in the hospital “doctor's lounge” appear to be a thing of the past, to the probable detriment of patient care. The “cradle to grave” family physician is a rare thing these days. More commonly, patients are quickly shuffled among various doctors and clinics, each attending to a symptom or disease problem rather than a patient. Important clinical information can be easily inadvertently omitted in such a scenario. Pap tests may or may not be sent to the same laboratory as the prior or subsequent biopsies, thus hindering cytologic–histologic correlations and lessening opportunities for clinical epiphanies. Finally, there is the anecdotal evidence that some laboratories may be reluctant to press submitting clinicians to provide complete clinical information on requisition slips, fearing loss of business volume in our current highly volatile health care climate.

In contrast to the seeming academic and clinical disinterest in the topic of reliability of data on requisition slips, trial lawyers are tuned in. Consider the following two exchanges from depositions of Plaintiff's experts:

Counsel: “There was a stipulation that all high-risk patients needed to go through the 10% re-screening process.” Expert: “The cytotechs…were relying upon only the requisitions coming with the slides from the clinicians and those are notoriously unreliable for history.” “Which is why the laboratories, modern laboratories, try to rely on their computer systems, or at least their data files."[4]

Counsel: “And so if, hypothetically, the patient had an abnormal biopsy reported out of another institution, unless the physician who's submitting the specimen tells you, is there any way that you are able to learn this information?” Expert witness: “In general, no.”…Counsel: “Do you profess to know what the standard of care is that applied to Dr…as a gynecologist when she filled out the requisition form?” Witness: Yes. She should have filled out the form; the patient had previous abnormal Paps. Counsel: “All right”…what you're telling me—correct me if I am wrong—is that there are standards of care that apply to gynecologists in this situation and that, in your opinion, Dr…deviated from the standard of care, is that correct?” Witness: “Yes, sir.”[5]

A prominent expert in the area of error reduction and risk management advised that “one stress the need for pertinent clinical history that is often required to initiate quality control measures for evaluation and reporting of cervical cytology specimens.”[6] Indeed, the solicitation of certain information is required by regulatory agencies and laboratory accreditation bodies (CMS (Centers for Medicare and Medicaid)/HHS (Health and Human Services)/CAP (College of American Pathologists)/TJC (the Joint Commission)/COLA) for good reasons, although laboratories cannot force clinicians to comply. One of the quality assessment standards that all these organizations recognize is that laboratories should actually do what they say they do in their laboratory policies and procedures.

Under CLIA '88, laboratories are required to include some high-risk cases in their mandatory 10% rescreen of slides initially screened as “negative for intraepithelial lesion or malignancy.” The definition of “high-risk” is left up to the individual laboratory. Some laboratories aim to rescreen all Pap test slides from “high-risk” patients, as defined by the laboratory. But, how does one know if one is accomplishing that goal?

The most important clinical finding of our study was that 77% of cases that would truly meet our laboratory's criteria for triage to a high-risk rescreen could in fact be accurately identified via the information provided on the requisition slips. However, there is room for improvement as 23% of Pap test slides from high-risk patients are potentially not being identified and rescreened as intended. For the purposes of this study, we reviewed all the LIS and Chartview data available to us. However, in the day-to-day practice, we routinely look only at the LIS records. The pathologists and, less often, the cytotechnologists only look up the Chartview medical records if there is a question at the time of reviewing the Pap. Resources do not permit us to perform intensive chart reviews on every routine Pap test that comes to the laboratory. Thus in real life, some of those patients in fact would be inadvertently denied an intended quality assurance rescreen, based upon our laboratory's quality assurance program.

We are a single laboratory. As this pre-analytical variable has not been previously measured, there are no benchmarks for it. We recognize that such a study would not even be feasible in many laboratories, which do not have access to corroborating data, as we do.

The completeness of Pap test requisition slips is one indicator of the effectiveness of laboratory to clinician communication. In the course of doing this study, we realized that we had not effectively communicated to the clinicians and their staff our expectations for the requisitions slips, and how we use them. A number of survey participants wrote us notes requesting the results and clarification concerning issues we raised. Anecdotally, we hear about laboratorians bemoaning the inaccuracy and incompleteness of requisition slips. And, here we see that our colleagues on the other end of the communication really do have an interest in doing what is necessary to provide the best outcome for their patients. So, it appears to us that this perceived universal problem is due to a breakdown in effective communication. Threats from regulators, government, and compliance officers or complaints from laboratorians can be expected to be nonproductive. However, here is an opportunity for interdepartmental cooperation and mutual education that can benefit all involved.

In practice, there are factors that limit optimal communications via Pap test requisition slips. One variable is how the information is obtained. For example, a patient may not remember the medically pertinent details of her past Pap test results or treatment. Improvement of laboratory-clinical communications, including design of requisition slips, optimization of clinical workflows, additional training and education of staff, and improved management of time constraints are possible areas of exploration for a future impact.

## CONCLUSIONS

Clinicians and their staff were very receptive to discussions of expectations of communications between them and the lab, and specifics on how those communications might impact and improve care for their patients. However, significantly, about half of them were unaware that the laboratory performed additional quality assurance rescreening of high-risk Pap test slides. Thus was identified a potential area for further education. Our initial version of the requisition slip did not elicit all the information sought by the laboratory, prompting revision. Clinical information and patient history were provided on the requisition slips in the vast majority of cases. The high accuracy of completion of requisition slips in this study permitted 77% of high-risk Pap tests to be identified via the requisition slip alone. Thus the common anecdotal impression of “notoriously unreliable”[4] information on Pap test requisition slips is not a universal truth. Because we are largely a contained health care system, our experience may not be applicable to other settings. The fact that 23% of high-risk Paps tests risk misclassification if classification is based upon the requisition slips alone indicates that there is room for improvement even in our setting.

## COMPETING INTEREST STATEMENT MADE BY ALL AUTHORS

No competing interest to declare by any of the authors.

## AUTHORSHIP STATEMENT BY ALL AUTHORS

All authors of this article declare that we qualify for authorship as defined by ICMJE http://www.icmje.org/#author.

Each author has participated sufficiently in the work and take public responsibility for appropriate portions of the content of this article.

Each author acknowledges that this final version was read and approved.

## ETHICS STATEMENT BY ALL AUTHORS

This study was conducted with approval from Institutional Review Board (IRB) (or its equivalent) of all the institutions associated with this study. Authors take responsibility to maintain relevant documentation in this respect.

## EDITORIAL / PEER-REVIEW STATEMENT

To ensure integrity and highest quality of CytoJournal publications, the review process of this manuscript was conducted under a double blind model [author(s) are blinded for reviewer(s) and reviewer(s) are/is blinded forauthor(s)] through automatic online system. CytoJournal is an open access platform to publish any peer-reviewed scientific point of view in Cytopathology and related areas. The views reflected in the article are those of authors alone and does reflect any agreement or disagreement by the journal and its publisher.
